# Plant Quantity Affects Development and Survival of a Gregarious Insect Herbivore and Its Endoparasitoid Wasp

**DOI:** 10.1371/journal.pone.0149539

**Published:** 2016-03-10

**Authors:** Minghui Fei, Rieta Gols, Feng Zhu, Jeffrey A. Harvey

**Affiliations:** 1 Netherlands Institute of Ecology, Department of Terrestrial Ecology, Wageningen, The Netherlands; 2 Laboratory of Entomology, Wageningen University, Wageningen, The Netherlands; 3 Department of Ecological Sciences, Section Animal Ecology, VU University, Amsterdam, The Netherlands; Charles University in Prague, CZECH REPUBLIC

## Abstract

Virtually all studies of plant-herbivore-natural enemy interactions focus on plant quality as the major constraint on development and survival. However, for many gregarious feeding insect herbivores that feed on small or ephemeral plants, the quantity of resources is much more limiting, yet this area has received virtually no attention. Here, in both lab and semi-field experiments using tents containing variably sized clusters of food plants, we studied the effects of periodic food deprivation in a tri-trophic system where quantitative constraints are profoundly important on insect performance. The large cabbage white *Pieris brassicae*, is a specialist herbivore of relatively small wild brassicaceous plants that grow in variable densities, with black mustard (*Brassica nigra*) being one of the most important. Larvae of *P*. *brassicae* are in turn attacked by a specialist endoparasitoid wasp, *Cotesia glomerata*. Increasing the length of food deprivation of newly molted final instar caterpillars significantly decreased herbivore and parasitoid survival and biomass, but shortened their development time. Moreover, the ability of caterpillars to recover when provided with food again was correlated with the length of the food deprivation period. In outdoor tents with natural vegetation, we created conditions similar to those faced by *P*. *brassicae* in nature by manipulating plant density. Low densities of *B*. *nigra* lead to potential starvation of *P*. *brassicae* broods and their parasitoids, replicating nutritional conditions of the lab experiments. The ability of both unparasitized and parasitized caterpillars to find corner plants was similar but decreased with central plant density. Survival of both the herbivore and parasitoid increased with plant density and was higher for unparasitized than for parasitized caterpillars. Our results, in comparison with previous studies, reveal that quantitative constraints are far more important that qualitative constraints on the performance of gregarious insect herbivores and their gregarious parasitoids in nature.

## Introduction

In nature, there is often considerable variation in the ways in which a species distributes itself within its habitat relative to conspecifics. Many organisms exhibit solitary lifestyles except when they are mating and/or raising their offspring. Still others live in groups of several to many thousands of individuals, although this may vary according to season. For group living to be favored, the overall benefits must outweigh the costs, and the resulting fitness pay-off to each group member must be greater than for a solitary individual [1]. In vertebrates, an advantage of group living is the development of social interactions among group members, which is often based on a shared genetic background [2]. Similar patterns may be observed within invertebrate groups, e.g. insects, where gregarious living is often based on the genetic relatedness of the individuals in the group [3]. This is certainly true in the social Hymenoptera, such as ants, bees and wasps, which reproduce via haplodiploidy and where distinct castes perform specific functions that optimize colony fitness [4]. Many phytophagous insects also lay their eggs in batches on their food plants and the immature (and sometimes adult) stages live in groups [5–7]. In the Lepidoptera (butterflies and moths), for example, large clutch size and aggregative feeding by the larvae has evolved independently in up to 23 families [8].

There are many obvious advantages of living in a group, (described as the Allee effect) which include the production of more offspring in a single reproductive event, faster development and more effective group-based defences against natural enemies [9–12]. However group living involves costs that include higher levels of disease transmission amongst closely assembled individuals [13, 14], and increased apparency to natural enemies that have the capability of attacking multiple prey [15]. Another important factor is that there is an increase in the level of intraspecific competition for potentially limiting food resources [12, 16–18]. Starvation of entire broods of insect herbivores and even population crashes may be mediated by the depletion of local food supply [19]. However, studies examining the evolution of gregarious development in insects rarely examine this parameter, and instead assume that later instars can always disperse to find new food plants once the natal plant is exhausted of tissues [12].

The vast majority of insect herbivores are specialized on plants producing phylogenetically conserved secondary metabolites [20]. Food deprivation is particularly problematical for immature feeding stages of gregarious specialist herbivores that feed on small, fast growing and/or ephemeral plants that are present for only a short time in the field. However, this constraint is rarely discussed in studies which instead focus primarily on plant quality as the major determinant of insect performance [21]. However, if the natal food plant is exhausted before the insects have reached maturity, then they are forced to disperse in search of new suitable plants, which may or may not be located nearby. This can have severe repercussions on their survival and/or fitness. For instance, when they are not feeding they expend metabolic energy, do not grow, and are thus susceptible to starvation and precocious death. Le Masurier (1994) reported that only 16% of final instar *Pieris brassicae* (cabbage butterfly) larvae located fresh host plants 2.5 meters from release sites over the course of 72 hours when they were released from a central point. For gregarious herbivores that produce large broods on small plants the mother should optimally choose plants that grow in large aggregations. However, eggs and larvae of gregarious herbivores like the peacock butterfly, *Inachis io* and large cabbage white butterfly, *Pieris brassicae* are occasionally laid on isolated plants and the larvae rapidly exhaust the plant and starve as a result.

Larval stages of Lepidoptera harbor many natural enemies, including parasitic wasps or ‘parasitoids’. These are insects whose larvae feed on, or in the host body whereas the adults are free living [22]. One group of parasitoids called ‘koinobionts’ attack hosts that continue feeding, growing and defending themselves during parasitism [23]. Solitary koinobiont parasitoids, in which only a single parasitoid larva develops inside the host, generally have lower nutritional requirements than gregarious parasitoids [23]. As a result, solitary parasitoids generally greatly reduce host growth, relative to that of unparasitized larvae, whereas gregarious parasitoids have little effect or may even stimulate increased host food consumption and growth [24]. Consequently, when a gregarious endoparasitoid attacks a gregarious-feeding herbivore host that in turn feeds on a small plant with insufficient biomass to support an entire brood, both organisms can potentially experience significantly negative effects on their survival and fitness. Thus far, however, this area has been little studied.

**Fig 1:**
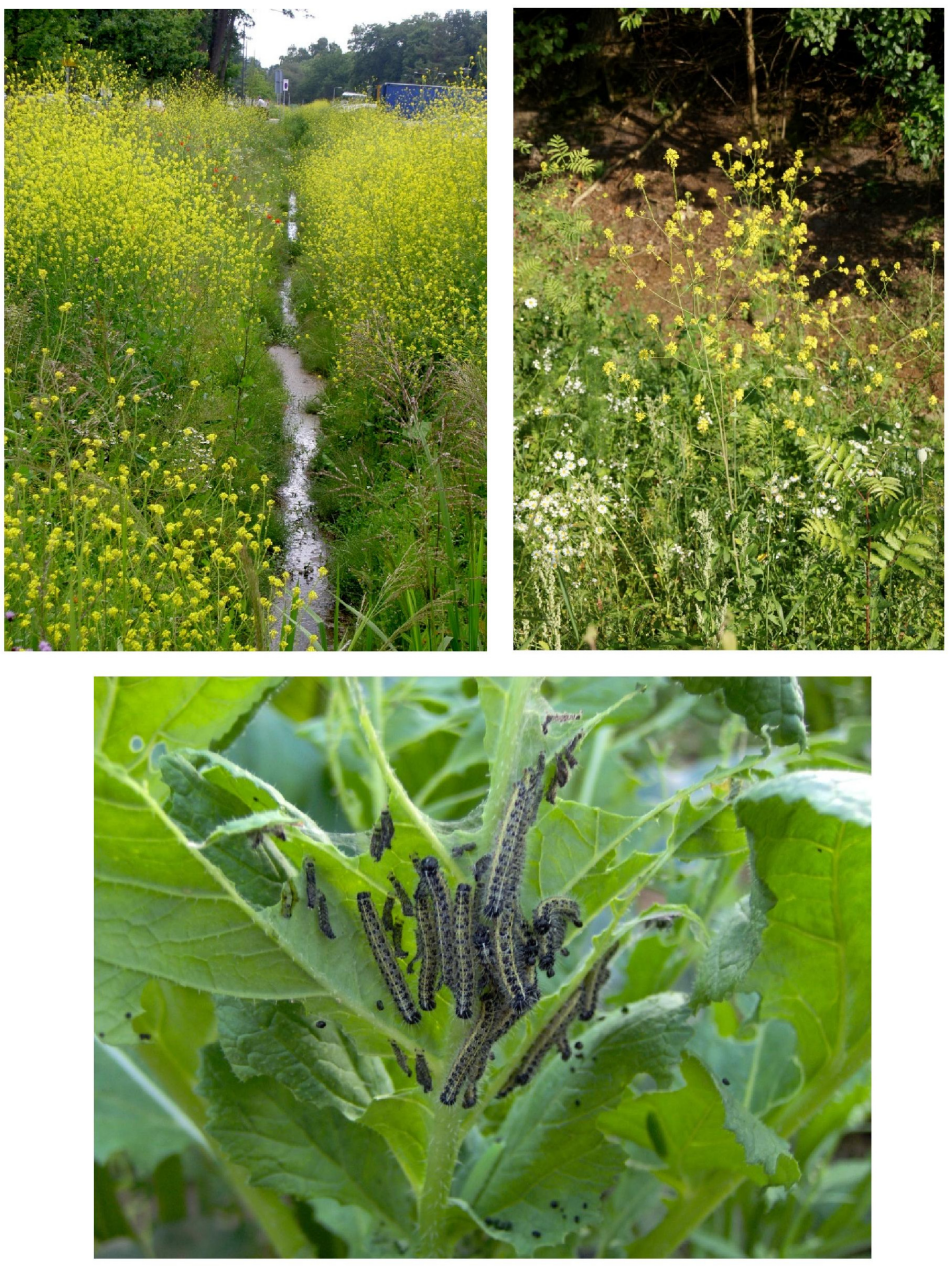
Clustered populations of *Brassica nigra* plants (upper left), a solitary plant (upper right) and feeding *Pieris brassicae* caterpillars (below).

Here, we examine development of the large cabbage white butterfly, *Pieris brassicae* L. (Lepidoptera: Pieridae) and its specialist gregarious endoparasitoid, *Cotesia glomerata* L. (Hymenoptera: Braconidae) under variable periods of food deprivation in the final instar. The final instar is critical for the development of most holometabolous insect herbivores, as 80% or more of larval biomass is accrued during this stage [25]. *P*. *brassicae* is a specialist herbivore of plants in the family Brassicaceae. One of the most important food plants in the Netherlands for *P*. *brassicae* is the black mustard, *Brassica nigra* (Brassicaceae), a small, fast growing annual weed, which grows along rivers and roadsides (Fig 1). *Pieris brassicae* lays broods of 30–150 eggs on the underside of mustard leaves and the larvae complete 5 instars before pupation. Instars 1–3 (hereafter L1-L3) feed in tight aggregations on the plant whereas the later instars disperse to some extent. *C*. *glomerata* females lay 20–40 eggs into L1-L2 *P*. *brassicae* larvae [24]. Caterpillars parasitized by *C*. *glomerata* are not killed by the parasitoid until the final instar, and often grow as large as unparasitized caterpillars [24].

In the field we have found *P*. *brassicae* broods growing on both isolated, solitary plants and plants growing in large populations (Fig 1). This tri-trophic system is both natural and a model system for studying nutrition-related constraints on herbivore (and natural enemy) survival and development. Thus far, studies with *P*. *brassicae* (with or without *C*. *glomerata*) have focused almost exclusively on plant quality effects on growth, development and survival [26–32] with only scant attention paid to plant size and/or quantity [5,7]. Indeed, *P*. *brassicae* has also been recently studied on *Arabidopsis thaliana*, where a single plant is too small to support the development of even a single caterpillar, let alone 50 or more [33–35]. Clearly studies ignoring quantitative constraints are lacking in ecological realism [36].

In lab experiments, we determined how periodic food deprivation of unparasitized and parasitized final-instar caterpillars affected survival and development (body mass and development time) of *P*. *brassicae* and *C*. *glomerata*. We also investigated the ability of herbivores and their parasitoids to recover from variable periods of food deprivation when reacquainted with plant diet. We then mimicked the starvation experiments in a semi-field experiment by placing parasitized and unparasitized caterpillars in groups of 50 onto plants grown in differing densities to determine how variation in plant biomass and natural barriers to dispersal–such as that provided by neighbouring vegetation–affects the survival of both parasitized and unparasitized larvae. We hypothesize that (1) food deprivation reduces herbivore and parasitoid survival and fitness, (2) both unparasitized and parasitized caterpillars are able to recover from food deprivation depending on the length of the food deprivation period, and that (3) in the field herbivore and parasitoid survival are negatively affected by a reduction in the density of focal plants, even when other mustard plants are placed nearby. As parasitism induces stress to the caterpillars, parasitized caterpillars will find new plants less successfully than unparasitized ones. We discuss our results in the context of food quantity and the spatial distribution of food plants as a major constraint on dietary breadth and development in gregarious insect herbivores.

## Materials and Methods

### Plants and insects

Specific permission was not required for collecting the *B*. *nigra* seeds which grow near Wageningen in the Netherlands, because they are wild plants and grow naturally in the field. We also confirm that the field studies in our experiments did not involve endangered or protected species. Seeds were collected from large populations of *B*. *nigra* which grow naturally along the River Rhine near Wageningen, The Netherlands (51°57'36.94"N, 5°40'45.87"O). Plants were grown from seeds in 1.1 L pots filled with peat soil (‘Lentse potgrond’ no. 4; Lent, The Netherlands) in a climate-controlled greenhouse at 21±2°C (day) or 16±2°C (night), 50% relative humidity, and 16h day length. If light dropped below 225 μmol photons m^-2^s^-1^ during the day, supplementary illumination was applied by sodium lamps. Plants were watered twice a week. When plants were 3 weeks old, they were fertilized weekly with Hoagland solution. Plants were approximately 4 weeks old at the start of each experiment, and were approaching maturity, with up to 10 mature leaves present per plant. Watering and fertilization continued during the experiments.

*Pieris brassicae* and *Cotesia glomerata* were obtained from a culture in Wageningen University, The Netherlands. *P*. *brassicae* were reared on Brussels sprout plants (*B*. *oleracea* var. *gemmifera*, cv. Cyrus) in a climate room at 22±2°C, 50–70% relatively humidity, and a photophase of 16:8D at Wageningen University (WU). Cultures of *C*. *glomerata* were reared on *P*. *brassicae* feeding on Brussels sprout plants, which were maintained under the same conditions as the host at the Netherlands Institute of Ecology (NIOO).

### General experimental protocol

To obtain *P*. *brassicae* caterpillars used in the experiments, over two successive days, 3-week old *B*. *nigra* plants were placed in the rearing cage with adult *P*. *brassicae* butterflies at the Entomology Department at WU for 24h. Plants containing egg clusters were removed from the butterfly cage and transferred to the experimental greenhouse. Once the eggs hatched the larvae were allowed to develop into the early 2^nd^ instar (L2).

Mated *C*. *glomerata* females used for parasitism originated from a culture maintained at the NIOO. Early L2 instar *P*. *brassicae* larvae were parasitized by presenting them individually to female parasitoids at the end of a small brush in a plastic vial. Previous studies [24, 26] showed that virtually 100% of stung caterpillars produce parasitoids (encapsulation does not occur).

As proxies for insect performance, we measured herbivore development time from egg hatching until pupation. Pupal fresh fresh mass was determined using a Mettler-Toledo (Columbus, OH USA) microbalance (accuracy±1μg). For the parasitoid, we determined the development time from oviposition to adult eclosion, adult fresh mass and secondary brood size (number of egressing parasitoid larvae that successfully spun cocoons). From each brood, a subsample of five males and or five females were weighed on the microbalance. Parasitoids were anaesthetized with CO2 before weighing.

#### Experiment 1. Effect of food deprivation duration of L5 *Pieris brassicae* on the performance of herbivores and parasitoids

Insect herbivores obtain 80% or more of biomass in the final instar, thus we examined temporal food deprivation in L5 *P*. *brassicae* on the performance of herbivores and parasitoids. Five cohorts of 150 1-day old L2 larvae were randomly selected, and each cohort was transferred to cages (40 x 40 x 55 cm) containing five *B*. *nigra* plants (five cages in total). In addition, five cohorts of 80 1-day old L2 larvae were randomly selected for parasitism by female *C*. *glomerata* wasps. Following parasitism, larvae were transferred in groups of 80 to cages containing five *B*. *nigra* plants (five cages in total). Unparasitized and parasitized caterpillars were allowed to move and feed freely on the plants within a cage. Additional plants were added when necessary. Larvae were reared in their respective cages with excess food until they molted into L5.

*P*. *brassicae* caterpillars were deprived of food at variable times during L5 beginning on the first day (S1 Protocol of Experiment 1). When caterpillars molted into L5 they were transferred to new cages in densities of 130 unparasitized or 60 parasitized caterpillars, with 5 separate cages for unparasitized caterpillars and 5 cages for parasitized caterpillars. Each cage contained 5 *B*. *nigra* plants. Five cohorts of 20 unparasitized and 8 parasitized from early L5 caterpillars were not transferred to new cages, but were instead placed in 9.5-cm Petri-dishes (1 caterpillar per dish) without food. On consecutive days thereafter 20 unparasitized caterpillars and 8 parasitized caterpillars were randomly taken from each of the cages and reared without food in Petri dishes (as above); this was continued until either pupation or parasitoid emergence in cages with food plants (S1 Protocol of Experiment 1).

The survival of both unparasitized and parasitized caterpillars in Petri dishes was recorded daily. The pupae of surviving caterpillars were weighed and the day of pupation was recorded. For parasitized caterpillars, when several adult wasps had emerged, the secondary brood size (number of cocoons) was recorded and as well as the development time to adult. At eclosion 5 male and 5 female wasps were also weighed on the microbalance.

#### Experiment 2. Insect recovery ability after variable food deprivation periods

As the field experiment shows, some caterpillars leave the natal plant when it is exhausted and find new plants nearby. Therefore, we set up an experiment to test the recovery ability of insects after variable food deprivation periods (S1 Protocol of Experiment 2).

Unparasitized and parasitized caterpillars were prepared as described for *Experiment 1* until molting into L5 and transferred into new cages. Most caterpillars in *Experiment 1* died when deprived of food during the first 3 days of their final (L5) instar. Therefore, three groups of 20 unparasitized and 15 parasitized caterpillars were randomly selected from the cages on the first day of L5 (4 replicates cages for unparasitized caterpillars, 5 replicates cages for parasitized caterpillars). These larvae were thereafter deprived of food for either 1, 2 or 3 days after which time they were once again provided with food plants. A second group of unparasitized caterpillars (4 replicate cages) was allowed to feed until day 3 since molting into L5. Then three groups of 20 unparasitized were randomly selected from the cages that were deprived of food for either 1, 2, or 3 days before they were re-provided with food plant. Because there were insufficient numbers of parasitized caterpillars, the second treatment was done with unparasitized caterpillars only (S1 Protocol of Experiment 2).

During the food deprivation period caterpillars were maintained individually in labeled Petri dishes (1 caterpillar per dish), and status (alive or dead) was monitored daily. After the food deprivation periods, insects from each replicate were placed in a new cage with 5 *B*. *nigra* plants. The survival and performance of these insects was followed until pupation of unparasitized caterpillars, or adult parasitoid eclosion. Performance parameters were measured using the same methods as described in *Experiment 1*.

#### Experiment 3. Effect of natal plant density on survival of of *Pieris brassicae* and *Cotesia glomerata* in semi-field experiment

To investigate the effect of food plant availability on survival of unparasitized and parasitized caterpillars, and on their ability to find a new food plant away from the natal patch, a semi-field experiment was conducted (for plant species composition see S1 Table) adjacent to the NIOO. Fifteen 3 x 3 m experimental plots were enclosed in meshed tents that prevented predation by birds (5 cm mesh). In the center of each tent potted plants were placed together in densities of 1, 5 or 8 *B*. *nigra* plants (5 replicates each), while in each corner of the tents 1.5 m away 2 potted *B*. *nigra* plants were placed.

Cohorts of 25 randomly selected 1-day old unparasitized and parasitized L2 larvae were transferred to each of 15 *B*. *nigra* plants. Caterpillars were kept on mustard plants in the greenhouse until they molted to L3, when they are too large to be successfully parasitized by *C*. *glomerata* [24]. Plants with L3 caterpillars were then placed in the center area of the tents. Plants in the corner of each tent were thereafter checked daily for *P*. *brassicae* caterpillars which were then removed from the plants and dissected in the lab to determine whether they had been parasitized or not. When caterpillars reached late L5 just prior to pupation, they were collected, both from center and corner plants, to ensure that they did not enter the wandering phase when they would be hard to find. The experiment was replicated three times in the summer of 2013 generating a total of 15 replicates per plant density (e.g. July to early September).

### Statistical analysis

For statistical analyses, cages (experiment 1 and 2) or tents (experiment 3) served as experimental units. The performance variables, development time and biomass, in experiment 1 and 2 were analyzed using a general linear mixed model (GLMM) where food deprivation period was entered as a fixed factor and cage as a random factor. The variance components were estimated using restricted maximum likelihood (REML) and the degrees of freedom in the approximated F-tests were adjusted according to the Satterthwaite method [37]. Tukey-Kramer multiple comparison tests between means were conducted when the model was significant.

For the parasitoid, the data were analyzed separately for males and females. Being gregarious, multiple *C*. *glomerata* individuals egress from a single caterpillar host. Thus, caterpillar host was included as a second random factor. The brood size has been demonstrated to affect the size/mass and development of the emerging adult wasps [24]. Therefore, brood size was included as a covariate in the model. The GLMM analyses were performed using the statistical package SAS version 9.3 (SAS Institute Inc., Cary NC, USA).

Logistic regression, i.e. a generalized linear model (GLM) was employed with a binomial distribution and logit link function, to analyze survival of unparasitized and parasitized caterpillars in relation to those subjected to variable periods of food deprivation. In the case of over-dispersion, we corrected for this by allowing the variance functions of the binomial distribution to have a multiplicative over-dispersion factor. The response variable in this analysis was the number of caterpillars that developed into pupae (unparasitized caterpillars) or produced parasitoid wasps (parasitized caterpillars) out of the total number of caterpillars that were initially collected for each food deprivation period. Caterpillar treatment (unparasitized or parasitized) was entered as a fixed factor and food deprivation period as a covariate. For the data on recovery ability following—food deprivation, we used a similar approach. In this analysis the time of initial food deprivation (early L5 or mid L5 caterpillar stages) was entered as a fixed factor and food deprivation period as a covariate (unparasitized caterpillars only). In a second analysis, we compared survival of unparasitized and parasitized caterpillars that were deprived of food as early L5 for variable time periods.

The data on survival in the semi-field experiment were also analyzed using logistic regression. We first investigated whether plant density of the central plant patch in the center of the tent had an effect on the proportion of the surviving caterpillars that were recovered on the additional plants placed in the corners of the tent at 1.5 meters distances from the central patch. As a measurement of overall survival, we also compared the recovery rate of the 25 released caterpillars either on the central or distant *B*. *nigra* plants. In both analyses caterpillar treatment was entered as a fixed factor and plant density as a covariate. Logistic regression analyses were performed in Genstat 17 (VSN International Ltd., Hemel Hempstead, UK).

## Results

### Experiment 1. Effect food deprivation duration of L5 *Pieris brassicae* on the performance of herbivores and parasitoids

#### Herbivore and Parasitoid Survival

**Fig 2:**
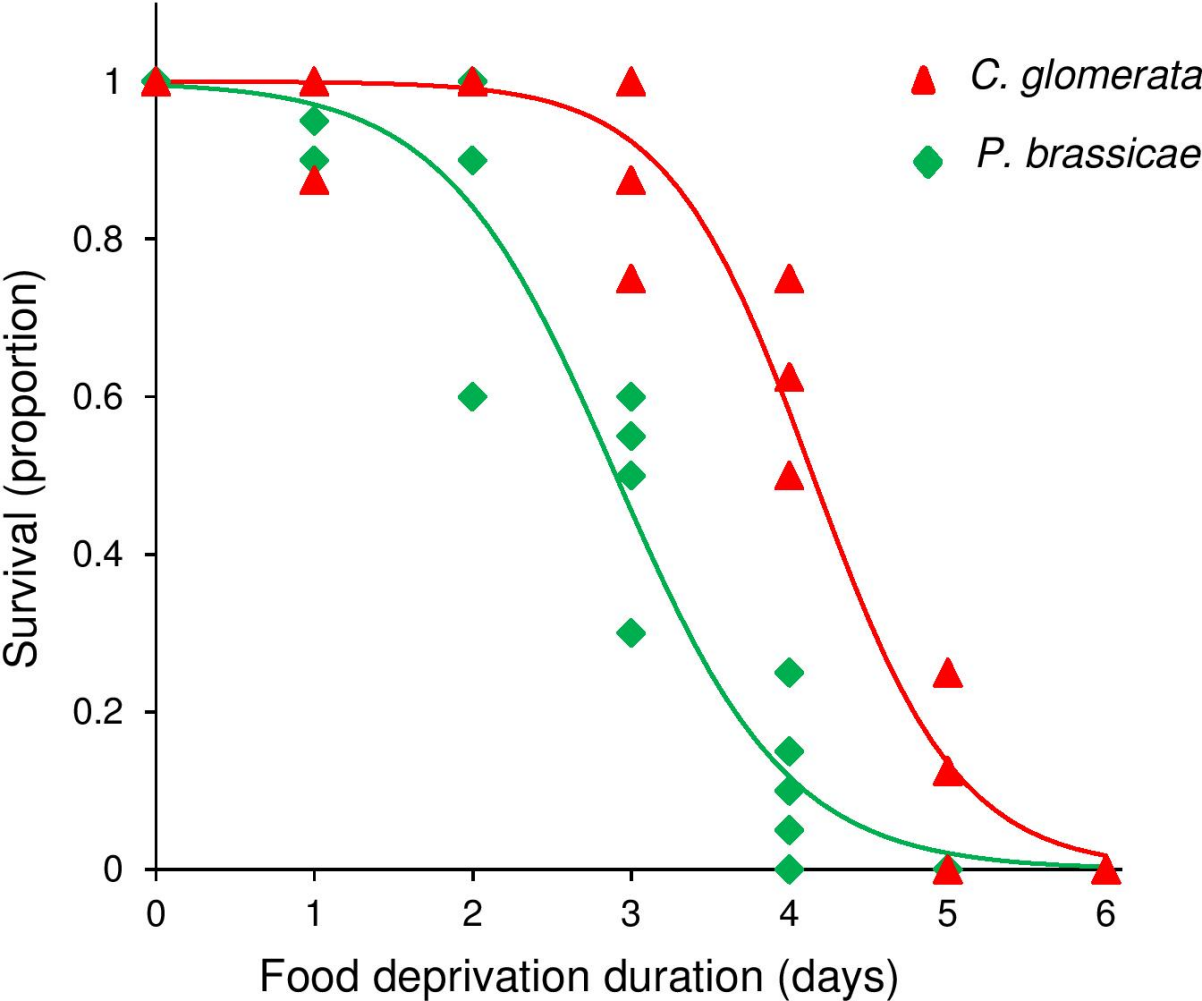
Effect of food deprivation duration of L5 *Pieris brassicae* on survival rate of the herbivore *Pieris brassicae* (green diamonds,) and the parasitoid *Cotesia glomerata* (red triangles,). Data are based on five replicates each with 20 unparasitized or 8 parasitized caterpillars.

Food deprivation duration dramatically reduced survival of the insects (GLM: χ^2^ = 717, df = 1, P < 0.001, Fig 2). The effect of food deprivation duration on survival was not significantly different for unparasitized *P*. *brassicae* caterpillars and those parasitized by *C*. *glomerata* (GLM: χ^2^ = 2.89, P = 0.09), neither was the interaction between density and species significant (GLM: χ^2^ = 1.21, P = 0.27).

#### Herbivore pupal mass and development time

**Fig 3:**
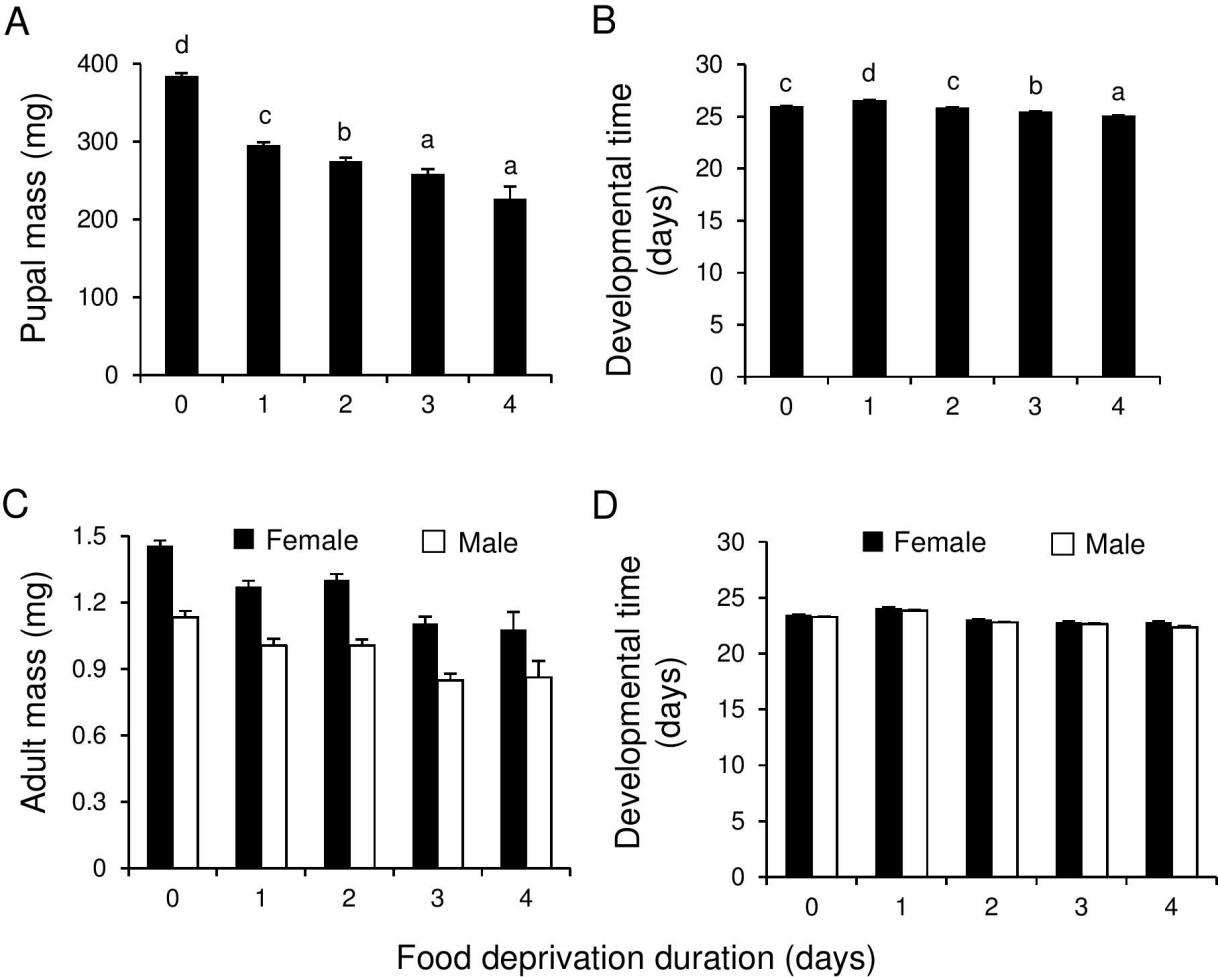
Effect of food deprivation of L5 *Pieris brassicae* on the performance of herbivores and parasitoids. Pupal fresh mass (A) and egg to pupa development time (B) of *Pieris brassicae*, and adult fresh mass (C) and egg to adult development time (D) of *Cotesia glomerata* females (black bars) and males (white bars). Bars (means + SE) with different letters are significantly different from each other (Tukey multiple comparisons). Sample sizes per cage (five cages per food deprivation treatment) were for *P*. *brassicae*: controls (constant food access): 20; deprived of food for one day: 18–19; two days: 12–20: three days: 6–11; four days: 0–5, and for *C*. *glomerata-*parasitized hosts: controls: 8; deprived of food for one day: 7–8; two days: 6–8; three days: 4–8; four days 3–6. For *C*. *glomerata*, adult fresh mass and development time was determined for the first 5 males and 5 females eclosing from each host cluster.

Food deprivation period had a significant effect on pupal mass of *P*. *brassicae* (F_4, 25.43_ = 134, P < 0.001) and development time (F_4, 18.54_ = 44.6, P <0.001). Pupal mass was negatively correlated with the duration of the food deprivation period, with the largest effect occurring on day before pupation (Fig 3A). Moreover, the longer the food deprivation period, the faster the larvae pupated (Fig 3B). However, the longest development time was found for caterpillars that were deprived of food one day prior to control caterpillar pupation (Fig 3B).

#### Parasitoid Adult Biomass and Development Time

Overall, the body mass of adult female wasps was higher (Fig 3C) and their development longer, than in male wasps (Fig 3D). However, the effect of food deprivation duration on adult mass also depended on the brood-size (GLMM brood size-food deprivation interaction: males F_4, 150_ = 2.94, P = 0.023: females F_4, 144.4_ = 3.27, P = 0.013). The length of the food deprivation period had a significant effect on adult fresh mass (GLMM males: F_4, 142.6_ = 11.6, P < 0.001: females F_4, 132.6_ = 14.2, P < 0.001). Similar as for unparasitized hosts, wasps weighed less the longer they were deprived of food (Fig 3C).

Food deprivation treatment, and brood size and their interaction had a significant effect on male development time (GLMM: treatment: F_4, 133.2_ = 2.60, P = 0.039; brood size: F_1, 153.6_ = 18.98, P < 0.001; interaction: F_4, 145.3_ = 3.10, P = 0.018). In general, development time decreased with brood size, however, the slope of this relationship depended on the food deprivation treatment. Here no consistent pattern could be discerned in relation to food deprivation duration. For females, only brood size had a significant effect on development time (F_1, 146.6_ = 8.69, P = 0.004), whereas both the effect of the food deprivation treatment and the interaction with brood size-treatment were not significant (GLMM: treatment: F_4, 133.3_ = 1.87 P = 0.12; brood size-treatment interaction: F_4, 143.7_ = 1.17, P = 0.33). As it is, there was no clear pattern for both male and female wasp development time with treatment (Fig 3D).

#### Parasitoid secondary brood size

**Fig 4:**
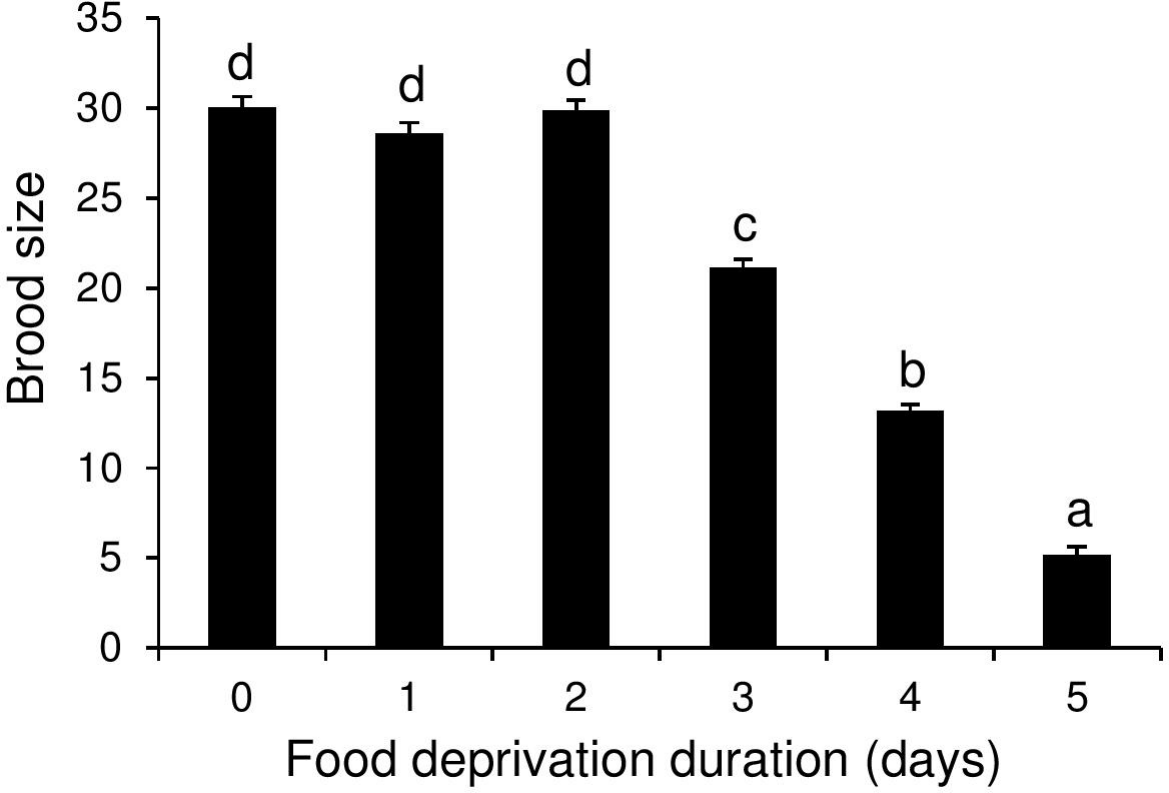
Effect of food deprivation duration of L5 *Pieris brassicae* on secondary brood size of *Cotesia glomerata*. Bars (means + SE) with different letters are significantly different from each other (Tukey multiple comparisons). Sample sizes were per cage (five cages per food deprivation treatment): controls (constant food access): 8; deprived of food for one day: 7–8; two days: 7–8; three days: 6–8; four days: 4–6, five days:1–2.

Food deprivation treatment also had a significant effect on secondary wasp brood size (F_5, 14.78_ = 41.98, P <0.001). Brood size decreased dramatically from approximately 30 in host caterpillars that were not deprived of food or were deprived of food for only 24–48 h prior to parasitoid larval egression to approximately 5 in host caterpillars that were deprived of food from the first day following the final molt (Fig 4).

### Experiment 2. Insect recovery ability after variable food deprivation periods of L5 *Pieris brassicae*

#### Herbivore Pupal Mass and Development time

**Fig 5:**
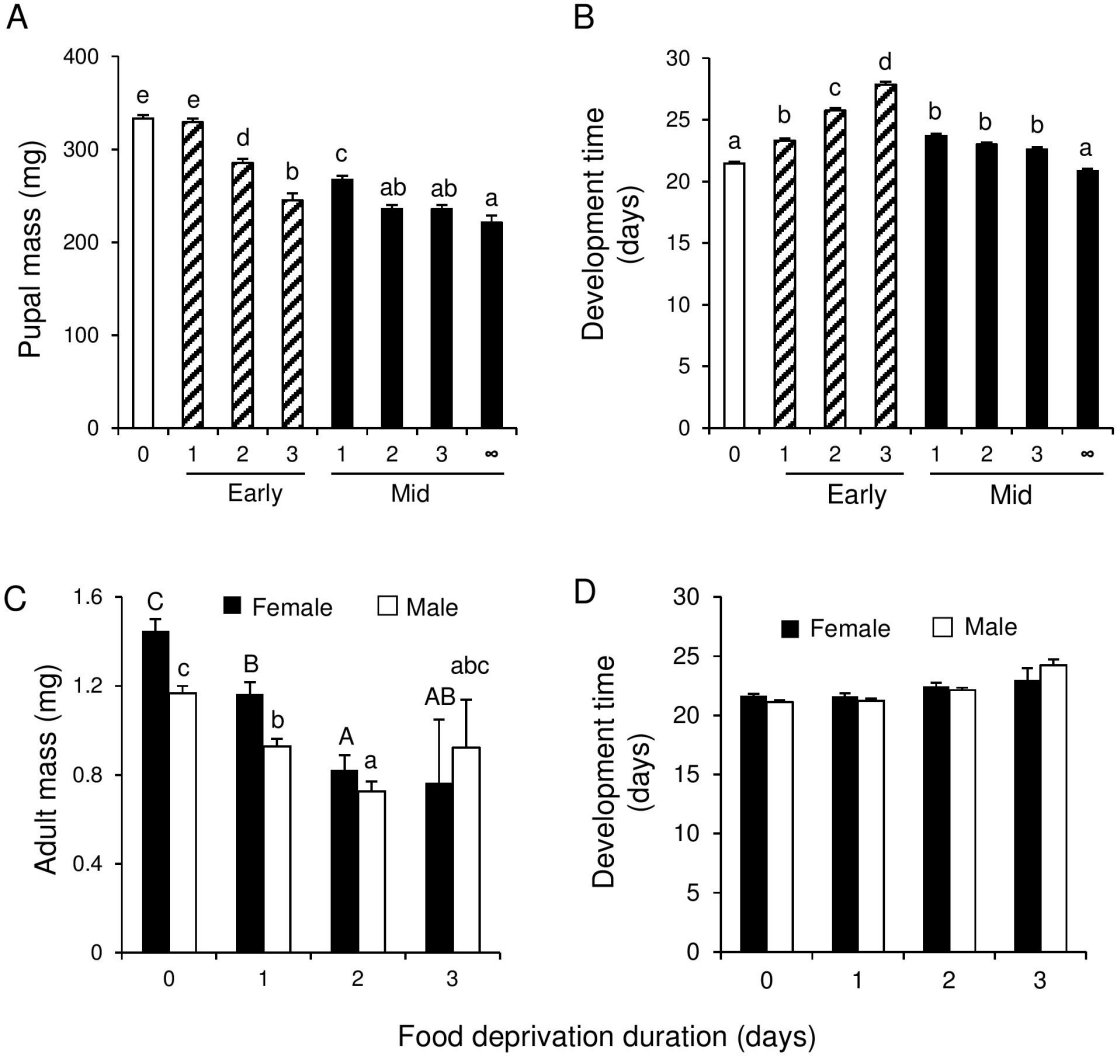
Insect recovery ability of of L5 *Pieris brassicae* after variable food deprivation periods. Pupal fresh mass (A) and egg to pupa development time (B) of *Pieris brassicae*, and adult fresh mass (C) and egg to adult development time (D) of *Cotesia glomerata* females (black bars) and males (white bars). Unparasitized *P*. *brassicae* caterpillars were deprived of food for variable periods from early L5 (dashed bars) or mid L5 (white bars), whereas hosts parasitized by *C*. *glomerata* were deprived of food only as early L5. Bars (means ± SE) with different letters are significantly different from each other (Tukey multiple comparisons). Sample sizes for *P*. *brassicae* were per cage (four cages per food deprivation treatment): controls (constantly fed): 20; deprived of food for one day as early L5: 20; two days as early L5:15–19; three days as early L5: 5–8; deprived of food for one day as mid L5: 14–20; two days as mid L5: 15–19;three days as early L5:15–18; food deprivation deprived of food as mid L5 until pupation: 5–7. Sample sizes for *C*. *glomerata* were per cage (five cages per food deprivation treatment): controls: 12–15; deprived of food for one day as early L5:9–14; two days as early L5: 3–12; three days as early L5: 1–3. For *C*. *glomerata*, adult fresh mass and development time was determined as the mean of the first 5 males and 5 females eclosing from each host cluster.

The ability to recover from food deprivation, depended on the duration of food deprivation both in terms of pupal mass (F_7, 29.74_ = 91.98, P < 0.001) and development time (F_7, 27.55_ = 26.4, P < 0.001). For pupal mass, this ability decreased more strongly with the duration of food deprivation for caterpillars that were deprived of food as early L5 compared to mid L5. However, caterpillars that were deprived of food for only one day when they were mid L5 did not fully recover from this, whereas caterpillars could fully recover that were deprived of food for one day when they were early L5 did (Fig 5A). When the food deprivation period was initiated one day following the final molt, development time increased with the length of the food deprivation (Fig 5B), whereas it was minor increased or even not affected when food deprivation was initiated when the larvae were already in the mid L5 stage (Fig 5B).

#### Parasitoid Adult Biomass and Development Time

The ability to recover from food deprivation in terms of adult fresh mass depended on food deprivation duration for both male and female wasps (GLMM: male: F_3, 79.35_ = 18.4, P < 0.001; female: F_3, 58.28_ = 8.79, P < 0.001) and was not affected by brood size (GLMM: male: F_1, 138.7_ = 0.34, P = 0.56; female: F_1, 94_ = 1.46, P = 0.23). Adult fresh mass of both sexes declined the longer the wasps were deprived of food before they were provided with food again, except for the male wasps with the longest (3 days) food deprivation periods (Fig 5C). Both male and female development time was longer in wasps that were deprived of food the longest before being provided with food again but this was statistically significant for the females (Fig 5D). For males, the ability to recover in terms of development was affected by food deprivation period (GLMM: F_3, 44.02_ = 26.3, P < 0.001) and brood size (GLMM: F_1, 122.1_ = 10.0, P = 0.002), but also by the interaction between brood size and food deprivation period (GLMM: F_3, 163.1_ = 4.68, P = 0.004). The development time of female wasps was neither effected by food deprivation period (GLMM: F_3, 31.23_ = 1.03, P = 0.39), brood size (GLMM: F_1, 70.5_ = 0.75, P = 0.39) nor was the interaction between these parameters significant (GLMM: F_3, 108_ = 0.22, P = 0.88).

#### Parasitoid secondary brood size

**Fig 6:**
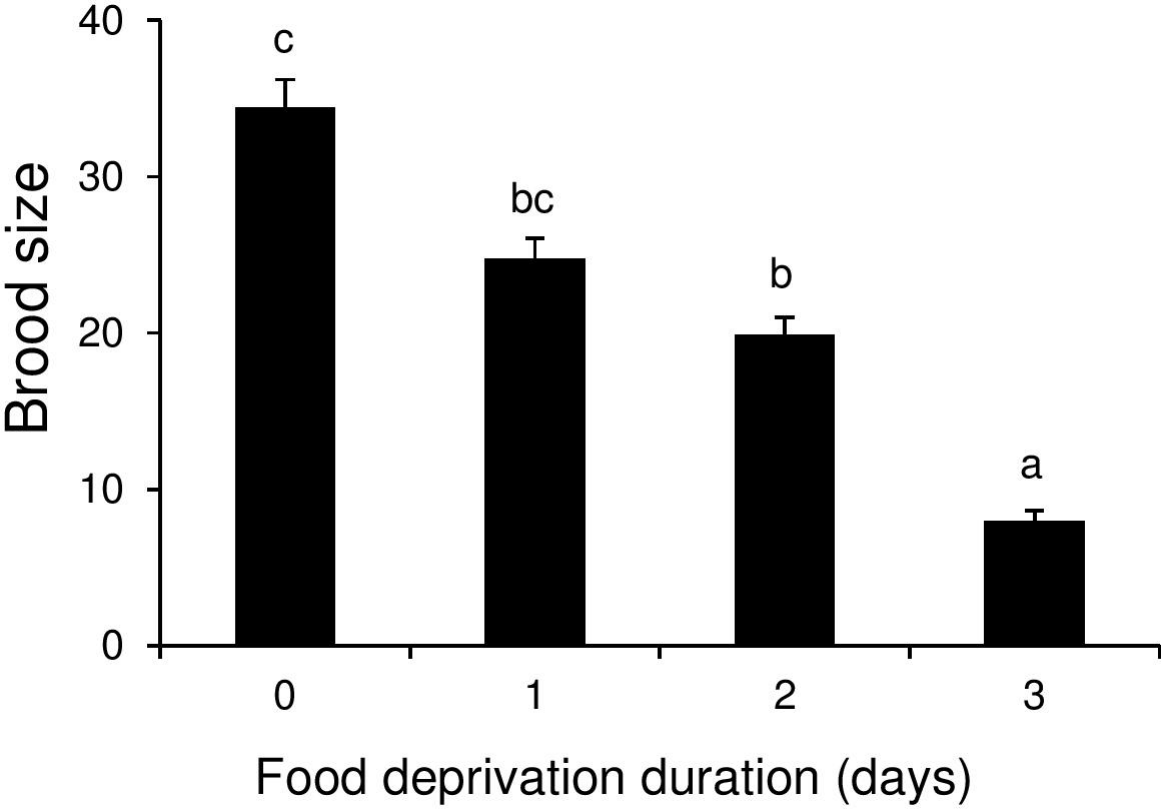
Effect of variable food deprivation periods of early L5 *Pieris brassicae* on secondary brood size of *Cotesia glomerata*. Bars (means + SE) with different letters are significantly different each other (Tukey multiple comparisons). Sample sizes were per cage (five cages per food deprivation treatment): without food deprivation controls: 14–15; deprived of food for one day: 12–14; two days: 4–12; three days: 1–3.

Parasitoid brood size was affected by the duration of host deprivation before hosts were re-provided with food (GLMM: F_3, 10.28_ = 15.4, P < 0.001) and decreased the longer that hosts were deprived of food (Fig 6).

#### Herbivore and parasitoid survival

**Fig 7:**
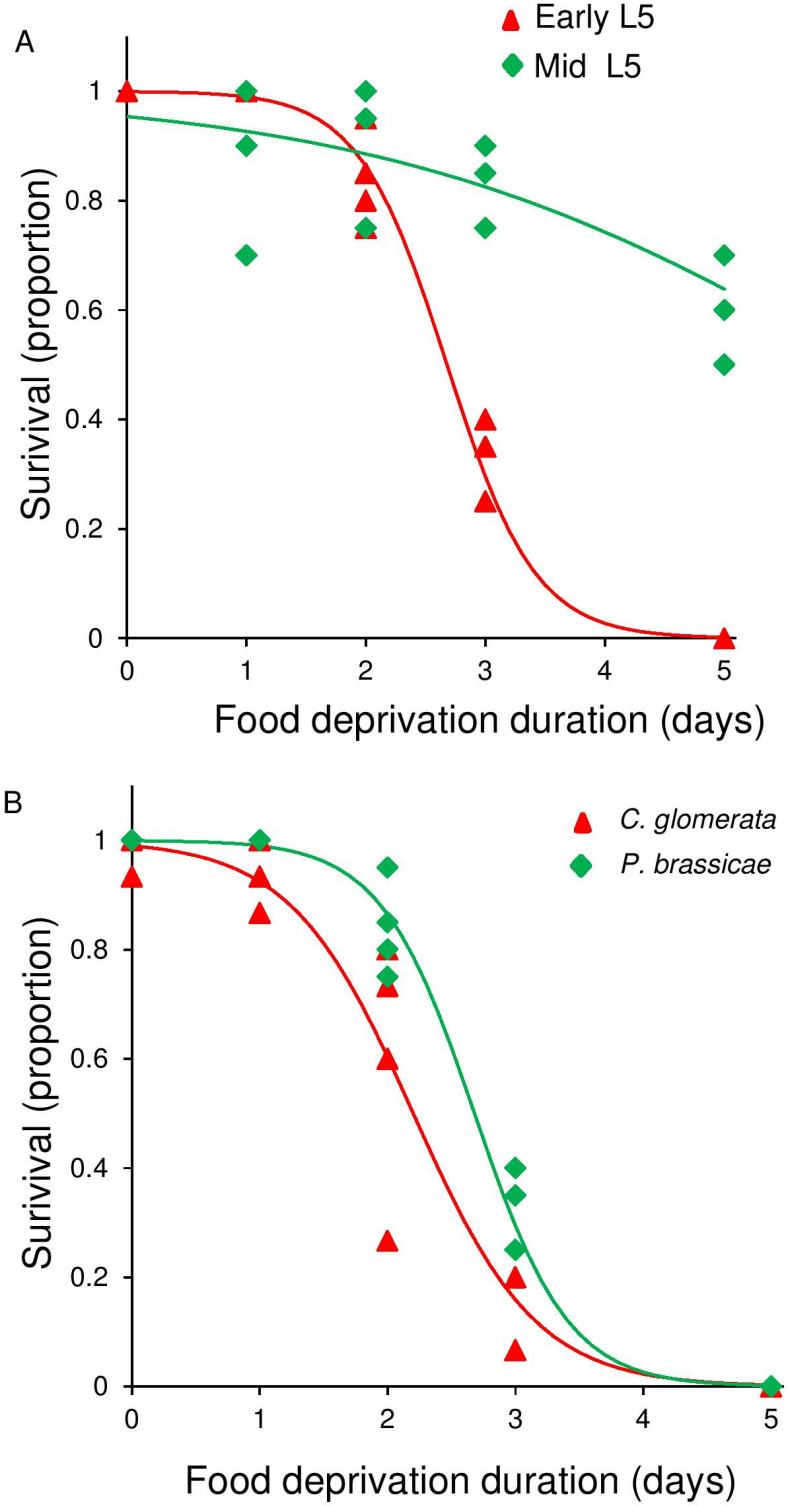
Effect of variable food deprivation period from early (red triangles) or mid (green diamonds) L5 on survival rate of *Pieris brassicae* (A) and effect of variable food deprivation period of early L5 *Pieris brassicae* (green diamonds) or *Cotesia glomerata* (red triangle) (B). Data are based on four replicates each with 20 unparasitized caterpillars and five replicates each with 15 parasitized caterpillars.

Food deprivation period affected recovery of unparasitized *P*. *brassicae* caterpillars that were deprived of food as early L5 more strongly that those deprived of food as mid L5 in terms of survival (GLM: onset of food derivation duration interaction: χ^2^_1_ = 76.5, P < 0.001; Fig 7A). Recovery of unparasitized and parasitized caterpillars that were deprived of food were similarly negatively affected by the length of the food deprivation period (GLM: species: χ^2^_1_ = 4.64, P = 0.031; food deprivation duration: χ^2^_1_ = 700, P<0.001; species- food deprivation duration interaction: χ^2^_1_ = 2.96, P = 0.085; Fig 7B). The survival rate decreased dramatically with the length of food deprivation (Fig 7B).

### Experiment 3. Effect of natal plant density on survival of unparasitized and parasitized caterpillars in semi-field set-up

**Fig 8:**
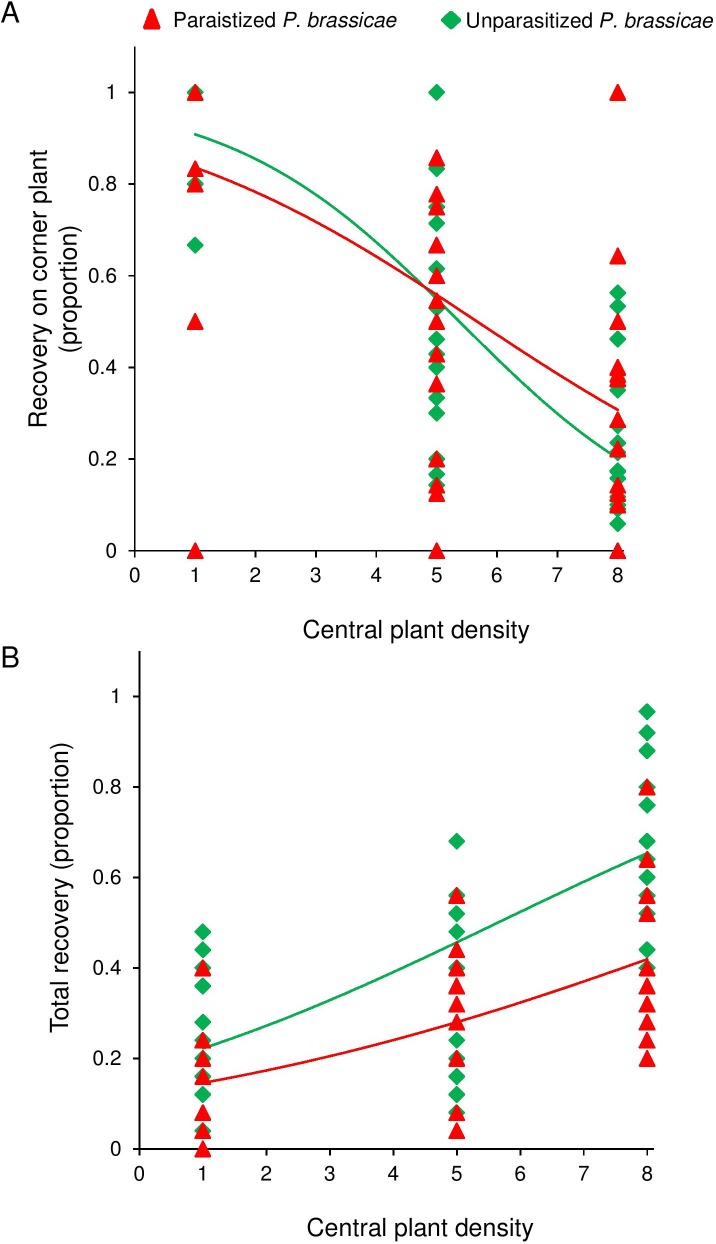
Relative recovery rate of unparasitized *Pieris brassicae* (green triangles) and parasitized *Pieris brassicae* (red triangles) on corner plants (A) with different center plant densities, and total recovery of *Pieris brassicae* (green diamonds) and *Cotesia glomerata* (red triangles) out of a total of 25 released parasitzed and 25 unparitized insects (B).

The density of the central food plant patch significantly affected dispersal to distant plants at 1.5 m distance (GLM: χ^2^_1_ = 104, P < 0.001) and this response was similar for unparasitized and parasitized caterpillars (GLM: species χ^2^_1_ = 0.88, P = 0.35, species-density interaction, χ^2^_1_ = 2.74, P = 0.10; Fig 8A). Fewer caterpillars were found on distant *B*. *nigra* plants (= corner plants) when the density of the central host plant patch (= center plants) increased. As an indication of mortality we also measured total recovery of the insects on any plant in the tent. The recovery of caterpillars increased with the density of plants in the central food patch (GLM: χ^2^_1_ = 67.2, P < 0.001) and was higher for unparasitized caterpillars than for the parasitized ones (GLM: species, χ^2^_1_ = 20.3, P < 0.001; species-density interaction, χ^2^_1_ = 1.04, P = 0.31; Fig 8B).

## Discussion

The results of this investigation show that depriving *P*. *brassicae* caterpillars of food for variable periods in the final instar significantly affected the survival and development of both the herbivore and its endoparasitoid, *C*. *glomerata*. Mortality of unparasitized caterpillars that were deprived of food one day after molting to the final (L5) instar was almost 100%, revealing that they did not reach a minimum viable size for pupation. However, when larvae were deprived of food for a shorter duration i.e. from 3^rd^ day of the final instar onwards, survival increased rapidly. Pupal mass was also negatively correlated with the duration of food deprivation. The final day of feeding is critical for *P*. *brassicae*, as larvae that were deprived of food for just one day prior to pupation were still some 25% smaller in terms of pupal mass than controls. By contrast, food deprivation had quite different effects on development time. Caterpillars that were deprived of food from the second day after molting to L5 developed significantly faster than those deprived of food for shorter periods or controls. On the other hand, larvae deprived of food just one day prior to control caterpillars took longer to develop than in any other treatment.

The effect of food deprivation on development in *C*. *glomerata* was similar to that of its host, with higher mortality, smaller brood size and smaller body mass of adult wasps developing in hosts deprived of food for longer periods. Larvae of *C*. *glomerata* feed primarily on hemolymph and fat body and consume about 80% of the fat content of their hosts [38]. As brood size increases, however, more fat is consumed, increasing potentially antagonistic interactions amongst siblings. A significant reduction in brood survival may have occurred through asymmetrical contest competition enabling a small number of ‘winners’ to exploit more *per capita* host resources. If competition had been more symmetrical amongst the siblings under scramble competition there would not have been enough food to facilitate survival of all siblings meaning all would have perished. Thuscontest competition amongst parasitoid siblings increases the chance that at least a few wasps survive, revealing some level of adaptation to resource-related constraints in *C*. *glomerata*. Starvation has been shown to affect the survival of other solitary endoparasitoids [39, 40] but few studies have examined how starvation affects gregarious endoparasitoids [41].

When unparasitized and parasitized caterpillars of *P*. *brassicae* were initially deprived of food for variable periods and then resupplied with *B*. *nigra* plants again, there were also significant effects on survival and development of both the herbivore and parasitoid. Caterpillars that were deprived of food for one day as early L5 almost totally recovered, but this declined sharply as the duration of food deprivation increased. Importantly, even short term food deprivation (e.g. 24 h) negatively affected insect performance, even when these individuals were given access to plants again. Thus, any kind of interruption in the feeding behavior of the herbivore can have significant fitness related effects on both itself and its endoparasitoid, and in the herbivore at least, these effects are correlated with the age (in L5) of the caterpillar.

In the semi-field experiments, we found that the density of clustered *B*. *nigra* plants significantly affected survival and dispersal of both unparasitized and parasitized *P*. *brassicae* caterpillars. The larger the density of centrally placed plants in tents the less likely the larvae were to emigrate in search of new mustard plants and the more that survived to late L5. The survival of parasitized caterpillars was somewhat lower then unparasitized caterpillars, suggesting that the former group is under greater physiological stress (e.g. nourishing two trophic levels instead of one) than the latter. Le Masurier [5] found that whereas 80% of released L4-L5 *P*. *brassicae* caterpillars located fresh cabbage plants placed 0.5 meters away from the release point, only 16% of larvae were able to locate fresh cabbage plants 2.5 meters away. However, in that study the larvae did not disperse voluntarily based on food availability but were released presumably as well fed caterpillars. Moreover, the background consisted of largely bare ground, which does not replicate a natural situation. In our experiments the caterpillars were allowed to choose when to leave the central plant(s) and had to navigate through dense stands of natural vegetation to reach new mustard plants 1.5 meters away. This vegetation not only represents a serious impediment to movement, but is also likely to harbor many natural enemies that pose a threat to dispersing caterpillars.

**Fig 9:**
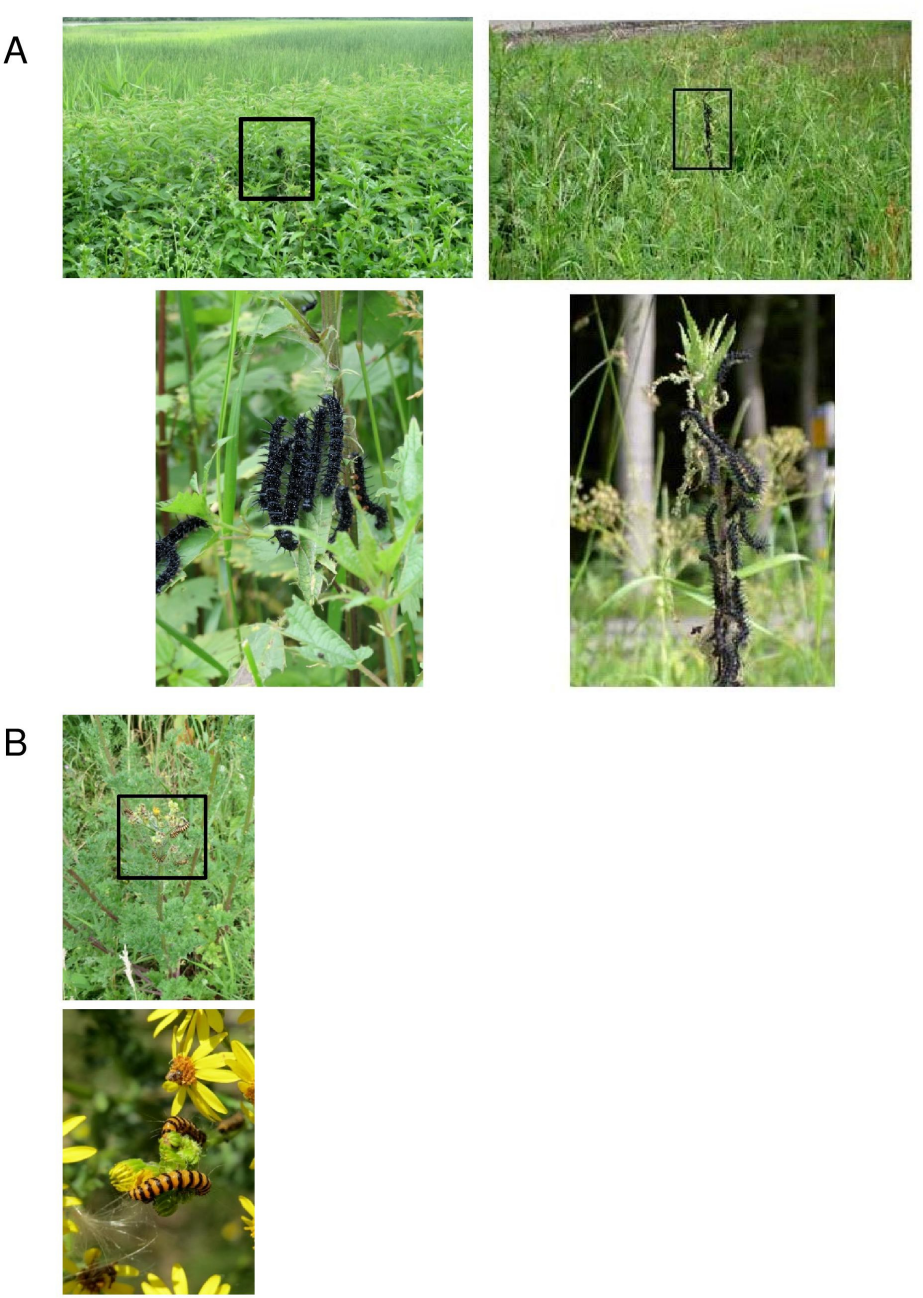
**(A)** Larvae of the Peacock Butterfly (inset and below), *Inachais io*, on aggregated plants (top left) and on an isolated plant (top right) of the Nettle, *Urtica dioica*. (B) Larvae of the Cinnabar Moth, *Tyria jacobaeae* (inset and below) on leaves of their food plant, the Tansy Ragwort *Jacobaea vulgaris*.

The progeny of gregarious butterflies developing on isolated plants may survive if nutritionally suitable plants are located in close proximity to the natal plant. However, there are both costs incurred as a result of energy expended in searching during the larval phase and an increase in the risk of predation when searching for new food plants. Moreover, if new plants are not located then the entire brood may starve. For example, starving L4-L5 caterpillars of the peacock butterfly, *Inachais io*, and *P*. *brassicae* have been observed in nature feeding on individual food plants, *Urtica dioica* and *B*. *nigra* respectively, that were tens of meters away from clustered plants of the same species (Fig 9A). The caterpillars had exhausted the natal plant. However, some gregarious Lepidoptera do adjust clutch size when choosing isolated plants with limited biomass. For instance, the cinnabar moth, *Tyria jacobaeae* (Fig 9B) typically lays no more than 10–15 eggs on tansy ragwort (*Jacobea vulgaris*) plants [42]. However, in this association, plants never grow in clusters but as individuals that are always separated from neighboring conspecifics by up to several meters. This suggests that spatial distribution of ragwort plants is predictable and has been a major selective force in the evolution of clutch size in the cinnabar moth. Interestingly, *P*. *brassicae* has been shown not to adjust its clutch size in the field [43].

Food limitation is clearly a potential threat to the survival and fitness of many gregarious-feeding insect herbivores and their parasitoids in nature, especially those associated with fast-growing, ephemeral plants like *B*. *nigra* and other weedy species. In much of Europe *P*. *brassicae* is restricted to feeding on wild brassicaceous plants that only grow in large enough clusters to support the development of a single brood [44]. This limits the number of quantitatively (but not qualitatively) suitable plant species on which they can develop and survive to around five. By contrast, the solitary congener *P*. *rapae* has been recorded on over 40 species of *Brassica* in Europe alone [45], including tiny species such as *A*. *thaliana*. This clearly shows that quantitative resource–related constraints on solitary specialist herbivores are considerably less than in gregarious species.

Thus far, most experiments studying plant-herbivore-natural enemy interactions in the lab as well as in the field select plants of optimal age and/or quality for the insects and, tacitly at least, assume that resources are not limiting [21]. Although broods of *P*. *brassicae* caterpillars depend on multiple plants growing together to ensure sufficient food is available to complete their development, focus thus far has been primarily on qualitative differences in plant tissues mediated by, for example, plant direct defence responses to herbivore damage [21, 29, 31, 32]. However, as we have shown here, the amount of food available to gregarious insect herbivores may be a much more serious constraint on their development and survival than quality. Optimality theory suggests that gregarious herbivores with large food requirements should always choose clustered plant resources or at least patches where the amount of food is more than necessary to support the successful development of the entire brood [46]. Studies have shown that clutch size decisions two two gregarious butterfly species are based plant quality and not size [47,48]. Neither study, however, measured constraints imposed by plant size and population structure on the development and survival of large broods. Similar conditions can also even occur on very large plants, such as trees, when insect herbivores occur in massive outbreaks. For example several moth species occasionally occur in outbreaks where numbers become so large that their larvae can even defoliate entire trees, leading also to starvation and potentially concomitant effects on their parasitoids [49–51]. However, a major difference is that these outbreaks are the result of many different mothers ovipositing on the same tree. In our study, we show that single broods may experience food shortage owing to limited biomass on the natal plant.

In summary, we have shown that food limitation exerts significant costs on the development and survival of a specialist butterfly and its endoparasitoid wasp. However, thus far the spatial arrangement (and/or size) of food plants and this affects herbivore survival and development has rarely been considered in empirical studies with gregarious herbivores and their specialist natural enemies. Moreover, limitation in food supply is not only based on plant density, but on other factors as well, such as age, and on the abiotic environment i.e. temperature, moisture etc. that may also affect plant quality [52]. We stress that future studies with both solitary and gregarious herbivores and their natural enemies–in particular, specialists—that are associated with small or ephemeral plants need to acknowledge the importance of quantitative constraints, and how these may be more important factors than quality in determining their success.

## Supporting Information

S1 Protocol of Experiment 1 (DOCX)

S1 Protocol of Experiment 2 (DOCX)

S1 Table Plant species of vegetation background in the semi-field experiment. (DOCX)
