# Supplementary material for: Plant Quantity Affects Development and Survival of a Gregarious Insect Herbivore and Its Endoparasitoid Wasp
Source: PLoS One. 2016 Mar 10;11(3):e0149539. doi: 10.1371/journal.pone.0149539 (PMC4786310; doi:10.1371/journal.pone.0149539)

**Protocol of experiment 1.** Unparasitized (A) and parasitized (B) *Pieris brassicae* caterpillars were deprived of food at progressing developmental stages during the final instar.


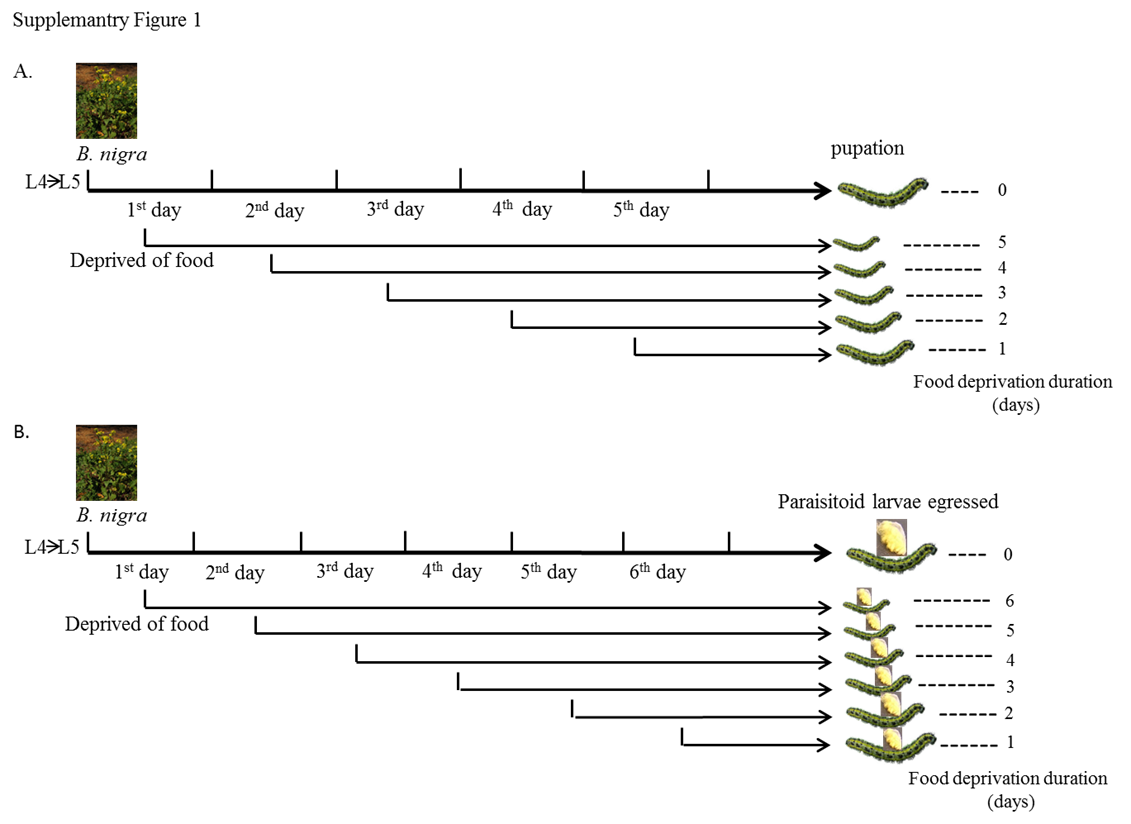

Supplement: S1 Protocol of Experiment 1 — (DOCX) [file pone.0149539.s001.docx]
