# Supplementary material for: Plant Quantity Affects Development and Survival of a Gregarious Insect Herbivore and Its Endoparasitoid Wasp
Source: PLoS One. 2016 Mar 10;11(3):e0149539. doi: 10.1371/journal.pone.0149539 (PMC4786310; doi:10.1371/journal.pone.0149539)

**Protocol of experiment 2**. Unparasitized (A, B) and parasitized (C) *Pieris brassicae* caterpillars were exposed to different food deprivation duration and then re-provided with food (*Brassica nigra* plant) from early of final instar (A, C) or mid of final instar (B).


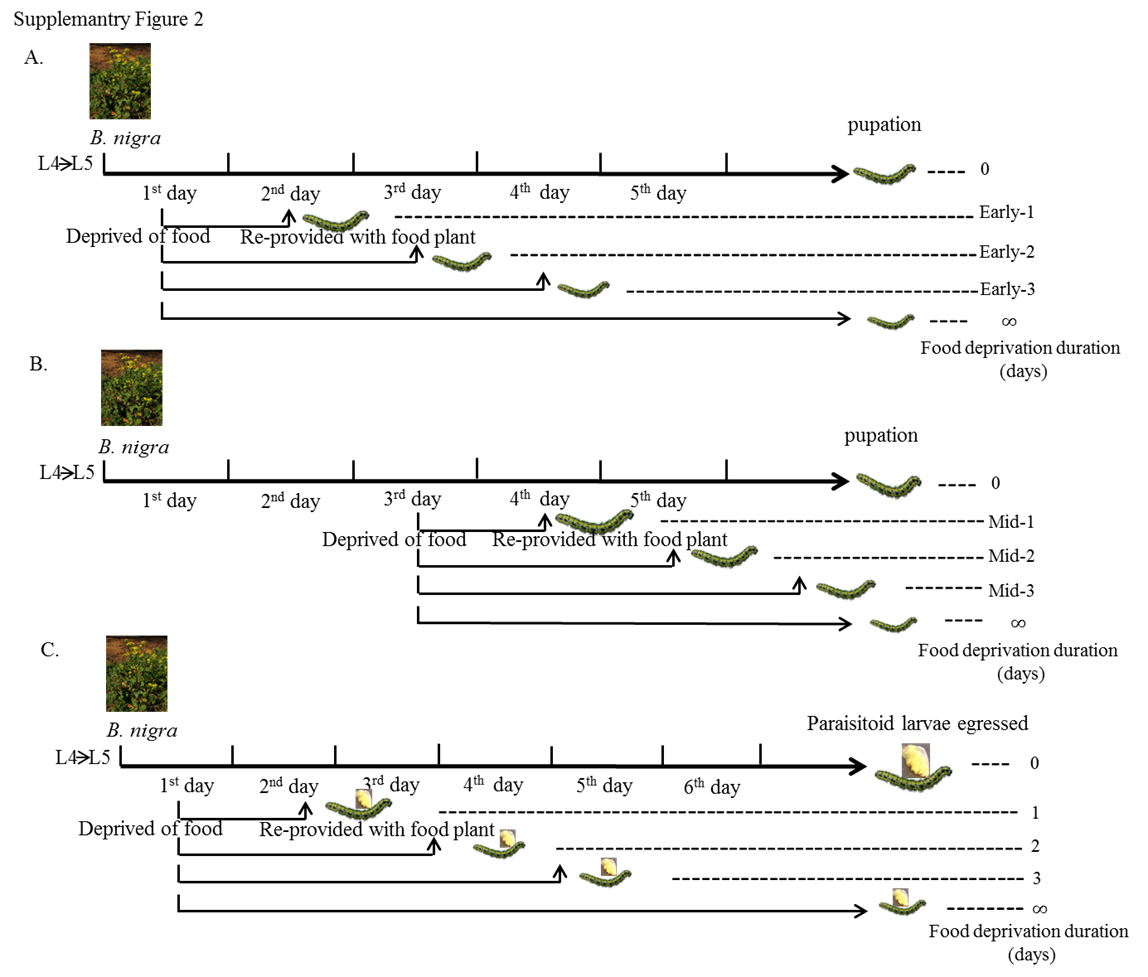

Supplement: S1 Protocol of Experiment 2 — (DOCX) [file pone.0149539.s002.docx]
