# Supplementary material for: Plant Quantity Affects Development and Survival of a Gregarious Insect Herbivore and Its Endoparasitoid Wasp
Source: PLoS One. 2016 Mar 10;11(3):e0149539. doi: 10.1371/journal.pone.0149539 (PMC4786310; doi:10.1371/journal.pone.0149539)
Supplement: S1 Table — (DOCX) [file pone.0149539.s003.docx]

**Table.** Plant species of vegetation background in the semi-field experiment

| 1 | *Plantago lanceolata* |
| --- | --- |
| 2 | *Trifolium repens* |
| 3 | *Taraxacum officinale* |
| 4 | *Tripleurospermum inodorum* |
| 5 | *Melilotus officianalis* |
| 6 | *Melilotus albus* |
| 7 | *Jacobaea vulgaris* |
| 8 | *Lolium perenne* |
| 9 | *Rumex acetosa* |
| 10 | *Festuca rubra* |
| 11 | *Cirsium arvense* |
| 12 | *Solidago gigantea* |
| 14 | *Ranucanlus acris* |
| 15 | *Conyza canadensis* |
| 16 | *Equisetum telmateia* |
| 17 | *Elipobium hirsutum* |
| 18 | *Urtica dioica* |
| 19 | *Tetragonolobus maritimus* |
| 20 | *Lythrum salicaria* |
| 21 | *Helichrysum orientale* |
| 22 | *Medicago sativa* |
| 23 | *Artemisia biennis* |
| 24 | *Ononis spinosa* |
| 25 | *Tephroseris integrifolia* |
| 26 | *Symphytum officinale* |
